# Dizocilpine Does Not Alter *NOS1AP* Gene Expression in Rats and in Cell Cultures

**DOI:** 10.3390/ijms26115329

**Published:** 2025-06-01

**Authors:** Anton B. Matiiv, Tatyana M. Rogoza, Irina A. Razgovorova, Maria I. Zhdanova, Nina P. Trubitsina, Mariya D. Bezgina, Irina G. Isaeva, Alexander G. Markov, Galina A. Zhouravleva, Stanislav A. Bondarev

**Affiliations:** 1 Department of Genetics and Biotechnology, St. Petersburg State University, 7/9 Universitetskaya emb., St. Petersburg 199034, Russia; 2 St. Petersburg Branch of the N.I. Vavilov Institute of General Genetics, St. Petersburg 199034, Russia; 3 Department of General Physiology, St. Petersburg State University, 7/9 Universitetskaya emb., St. Petersburg 199034, Russia; 4 Laboratory of Amyloid Biology, St. Petersburg State University, 7/9 Universitetskaya emb., St. Petersburg 199034, Russia

**Keywords:** NOS1AP, dizocilpine, MK-801, NMDA, schizophrenia, wistar rats, SH-SY5Y

## Abstract

The *NOS1AP* gene encodes the nitric oxide synthase 1 adaptor protein (NOS1AP), which binds to neuronal nitric oxide synthase (nNOS) and regulates nitric oxide (NO) production by dissociating nNOS from NMDA receptors (NMDARs). Notably, NOS1AP expression is upregulated upon NMDAR activation; however, there is no available data regarding its production under the receptor inhibition. The *NOS1AP* gene is also 1 among more than 1000 genes that are presumed to be associated with the development of schizophrenia. Various animal models of this disorder have been developed, some of which are based on the use of NMDAR antagonists such as dizocilpine (MK-801). In this study, we investigated the expression and production of NOS1AP in rats injected with a low dose of dizocilpine (0.1 mg/kg), as well as in SH-SY5Y and HEK293T cell lines treated with varying concentrations of the same compound (10–200 µM). According to our results, neither the expression of the *NOS1AP* gene nor the production of NOS1AP protein was affected by dizocilpine treatment.

## 1. Introduction

The *NOS1AP* gene encodes a nitric oxide synthase 1 adaptor protein (NOS1AP). It binds to neuronal nitric oxide synthase 1 (nNOS or NOS1) and is therefore involved in the regulation of nitric oxide (NO) production. The binding of NOS1 by NOS1AP is supposed to alter the localization of the synthase and prevent its interaction with NMDA receptors (NMDAR). As a result, the enzyme cannot produce NO in response to Ca^2+^ influx [1]. Thus, NOS1AP can be considered as a part of the NMDA signaling.

NOS1AP contains 506 amino acid residues and comprises two domains: an N-terminal phosphotyrosine-binding domain (PTB) and a C-terminal PDZ-binding domain (PDZ-BD) [2]. Several alternative transcripts of *NOS1AP* have been described [3,4]. NOS1AP interacts with nNOS via its PDZ-BD. Through the PTB domain, NOS1AP binds to Dexras1, resulting in the formation of a ternary protein complex composed of NOS1AP, Dexras1, and nNOS, which is required for nNOS to activate Dexras1 [5]. Through the PTB domain, NOS1AP also interacts with synapsins I, II, and III to form a complex required for the localization of nNOS to presynaptic terminals.

*NOS1AP* is 1 of more than 1000 candidate genes identified as putatively associated with the development of schizophrenia (according to the SZGene database [6]). This gene is located on the long arm of chromosome 1 (locus 1q23.3), and several genetic studies have supported the potential role of the nearby region in the development of schizophrenia. Linkage has been found at the following loci: 1q21-q22 (Canadian families of Celtic and German descent [7] and families from the USA, UK, Ireland, Northern Italy, and Belgium [8]), 1q22 [9], 1q22-23 (British and Icelandic families [10]), and 1q (Taiwanese families [11]). Finally, the fine mapping revealed linkage specifically with the *NOS1AP* gene [12].

Investigating the functional role of NOS1AP in schizophrenia development is challenging in humans. However, the analysis of post-mortem samples of patients from different regions of the USA has revealed an increased level of the *NOS1AP* gene mRNA as well as the corresponding protein. Moreover, the expression level of *NOS1AP* was found to correlate with single-nucleotide variations (SNVs), which have been shown to be associated with schizophrenia in Canadian families [13]. From another point of view, healthy individuals with schizophrenia-associated SNVs in *NOS1AP* demonstrated significantly greater activation of the dorsolateral prefrontal cortex when performing a task of working memory [14].

Despite extensive research, the molecular mechanism of schizophrenia is not fully elucidated. There are at least two hypotheses on the pathogenesis of this disorder: the glutamate (for review see [15]) and the dopamine (for review see [16]) hypotheses. According to the former, the hypofunction of NMDAR is a key mechanism. NOS1AP overproduction described in patients with schizophrenia [13] is in good agreement with this hypothesis because it may prevent the interaction between NOS1 and NMDAR and thus decrease the activity of the receptor.

It was previously shown that the activation of the NMDA pathway increases NOS1AP production [17]. At the same time, the NMDAR antagonist dizocilpine (MK-801) is widely used in various animal models of schizophrenia (for a review, see [18]). The effect of MK-801 treatment on NOS1AP expression has not yet been studied. We therefore aimed to fill this gap by investigating the effect of the NMDAR inhibitor MK-801 both in an animal model and in vitro.

## 2. Materials and Methods

### 2.1. Animals with Symptoms of Schizophrenia

To obtain animals with symptoms of schizophrenia, Wistar rats (aged 13–14 weeks) were subjected to a series of dizocilpine injections according to the previously described methodology [19]. Dizocilpine injections occurred from 13:00 at 5 min intervals. Laboratory rats weighing 215–270 g were purchased from Rappolovo breeding nursery (NRC “Kurchatov Institute”, St. Peterburg, Russia). The animals were kept in a room with controlled temperature and humidity, 12:12 h light–dark cycle, and had free access to food and water. Male rats (m = 270 g) were used to produce symptoms of schizophrenia. The experimental group was intraperitoneally injected with dizocilpine solution (M107-50MG, Sigma-Aldrich Inc., MilliporeSigma, St. Louis, MO, USA) for 6 days at the rate of 0.1 mg per 1 kg of body weight [19]; the control group received equivalent volume of sodium chloride solution (0.9% NaCl). On the seventh day, the animals were taken in the circular open field test (RPC OpenScience Ltd., Krasnogorsk, Russian Federation) The test time was chosen by taking into account the conducted injections and daily activity of rats during daylight hours from 14:00 [20]. Further, the animals were decapitated and their brains were extracted. The extracted brain fragments included the medial prefrontal cortex, hippocampus, and striatum (NOS1AP is produced in these parts [21,22]). For additional analysis, the listed brain parts were also isolated independently. Samples for protein isolation were frozen in liquid nitrogen; material for RNA isolation was also placed in RNA stabilization solution (RNAlater™, AM7020, Invitrogen™, Thermo Fisher Scientific Inc., Waltham, MA, USA). All samples were stored at −80 °C until the start of experiments.

### 2.2. Treatment of Cells with MK-801

Two cell lines were used: SH-SY5Y neuroblastoma cell line and HEK293T. Neuroblastoma cells were grown on DMEM/F12 medium supplemented with fetal bovine serum (FBS) up to 15% (*w*/*v*); HEK293T was grown in DMEM medium with 10% FBS. All media contaned penicillin and streptomycin at final concentrations of 100 units/mL and 100 µg/mL, respectively. Cells were cultured at 37 °C in an atmosphere of 5% CO_2_.

For all experiments, cells were seeded at a density of of 5 × 10^5^ cells in 2 mL of appropriate medium in one well of a 6-well plate. After 48 h, MK-801 was added to the cells in different concentrations. Saline was added as a control. For RNA isolation, cells were washed off the dishes with trypsin-Versen solution (Biolot Ltd., St. Petersburg, Russian Federation) after 6 or 48 h of incubation. Previously, it was shown that 6 h is sufficient to detect changes in NOS1AP expression [17]. Cells were then precipitated by centrifugation at 1200× *g* for 5 min at 4 °C, washed with cold PBS, and stored at −80 °C. For protein isolation, cells were collected in the same way 24 or 48 h after incubation.

### 2.3. RNA Isolation

To estimate the alteration of *NOS1AP* gene expression by qRT-PCR, total RNA was isolated using an RNA isolation kit (GeneJET™ RNA Purification Kit, K0731, Thermo Scientific™, Thermo Fisher Scientific Inc., Waltham, MA, USA). The quality of isolated RNA was monitored by agarose gel electrophoresis. In the following step, total RNA was purified from genomic DNA using Dnase I (RapidOut DNA Removal Kit, K2981, Thermo Scientific™, Thermo Fisher Scientific Inc., Waltham, MA, USA)and cDNA was synthesized using a reverse transcription kit (RevertAid™ RT Kit, K1691, Thermo Scientific™, Thermo Fisher Scientific Inc., Waltham, MA, USA) with oligo dT primers. RNA isolation from animal brains and cDNA synthesis were performed similarly to experiments with human neuroblastoma cells.

### 2.4. Protein Extraction

The protein extraction was performed as described previously [23], with minor modifications. An equal volume of RIPA buffer (150 mM NaCl, 1% (*w*/*v*) Triton X-100, 0.5% (*w*/*v*) sodium deoxycholate, 0.1% (*w*/*v*) SDS, 50 mM Tris-HCl (pH 8.0), 2 mM PMSF, 10 µg/mL leupeptin, 20% (*v*/*v*) protease inhibitors (P8340-5ML, Sigma-Aldrich Inc., MilliporeSigma, St. Louis, MO, USA)) was added to SH-SY5Y cells. Cells were incubated in buffer for 30 min on ice. The solution was then sonicated (10 s at 50% power with a SONOPULS HD 2070 (BANDELIN electronic GmbH & Co. KG, Berlin, Germany) ultrasonic homogenizer). The cell debris was collected by centrifugation at 2000× *g* at 4 °C for 10 min. The supernatant was used to prepare samples for SDS-PAGE.

Sodium deoxycholate buffer (50 mM Tris-HCl (pH 9.0), 1% (*w*/*v*) sodium deoxycholate, 20 µM ZnCl_2_, 0.5 mM PMSF, 2 µg/mL leupeptin) was used to isolate proteins from rat brains. Brain tissues were minced with liquid nitrogen; buffer was added at a ratio of 18 mL buffer per 1 g brain and the mixture was incubated for 1 h on ice. Cell debris was collected by centrifugation at 2000× *g* at 4 °C for 30 min. The supernatant was used to prepare samples for SDS-PAGE.

### 2.5. qRT-PCR

The PCR reactions were performed using a qRT-PCR C1000 Touch Thermal Cycler and CFX96 Optical Reaction Module (Bio-Rad Laboratories, Inc., Hercules, CA, USA), where the total volume per reaction was 20 µL, containing 20 µM of each primer NOS1AP_1&3i_F and NOS1AP_R (allow to amplify fragments of long *NOS1AP* transcripts) or reference primers to the gene β-actin or ribosomal protein lateral stalk subunit P0 (*RPLP0*) (Table 1), the cDNA (dilution 1:10 or 1:2.5, depending on RNA concentration), and 2.5× Eva Green Reaction mix for qRT-PCR (M-439, Syntol, Moscow, Russian Federation). Before experiments, we estimated and compared the PCR efficiency for all primer pairs (Table 1).

Reactions were performed with thermocycling parameters possessing a pre-incubation of 5 min at 90 °C, followed by a three-step amplification program of 30 cycles consisting of a denaturation, annealing, and extension step set at 94 °C for 30 s, 50/60 °C for 30 s, and 72 °C for 30 s, respectively. Then, the final step of an extended elongation period was 72 °C for 5 min. Reactions with reference primers to the genes β-actin or *RPLP0* were performed using 55.7 °C or 62.0 °C annealing temperature, respectively. The efficiency of *NOS1AP* amplification did not change under such conditions. The further analysis and the determination of relative quantity and normalized relative quantity (NRQ) was performed according to previously published recommendations [25].

### 2.6. SDS-PAGE and Western Blotting

Electrophoresis was performed according to standard methods [26,27] at 180 V for 50–60 min. A Trans-Blot Turbo rapid transfer system (Bio-Rad Laboratories, Inc., Hercules, CA, USA), Hybond^®^ P 0.45 PVDF blotting membrane (Sigma-Aldrich Inc., MilliporeSigma, St. Louis, MO, USA), and Whatman^®^ gel blotting paper (3030-672, GE Healthcare Technologies Inc., Chicago, IL, USA) were used to transfer proteins to the membrane according to the method in [28]. The transfer was carried out at 25 V for 30 min.

Western blot hybridization was performed as described [29]. Anti-NOS1AP antibodies [30] were used for NOS1AP detection, and anti-rabbit IgG antibodies (NA934-1ML, Cytiva, Global Life Sciences Solutions USA LLC, Marlborough, MA, USA) were used as secondary antibodies. For tubulin detection, Anti-α-Tubulin (Sigma, T6074) was used as the primary antibody, and anti-mouse IgG antibodies (NIF825, Cytiva, Global Life Sciences Solutions USA LLC, Marlborough, MA, USA) were used as secondary antibodies. Chemiluminescence detection was performed on a GeneGnome imaging System (Syngene, Bangalore, Karnataka, India).

Image processing after Western blot was performed using ImageJ v. 1.54g [31]. Hybridization intensity analysis was performed by hand using the ImageJ software package. We evaluated the chemiluminescence intensities of NOS1AP and α-Tubulin protein signals and then calculated the ratio of NOS1AP protein signal to α-Tubulin signal.

### 2.7. Statistical Analysis

All statistical processing, as well as plotting, was performed using the R-4.3.2 package [32]. The packages “ggplot2” and “plyr” were used for data visualization [33,34]. Quantitative comparisons were performed using the Wilcoxon–Mann–Whitney test [35] with multiple comparison adjustment (Holm–Bonferroni method).

## 3. Results

MK-801 is an NMDA receptor antagonist, and the injection of this drug is one method for producing symptoms of schizophrenia in animals. It was previously shown that injections of high doses of MK-801 (0.5 mg/kg) led to various behavioral abnormalities such as hyperlocomotion, ataxia, abduction of the hind limbs, and stereotyped behavior in rats [36]. Smaller doses of MK-801 (0.1 mg/kg) were shown to induce behavioral changes in rats, but not neurochemical alterations seen in schizophrenia. At the same time, this model allows the investigation of NMDA hypofunction in vivo [19]. It has previously been shown that the NMDA treatment of astrocytes can increase NOS1AP production [17]. This fact revealed that the production of NOS1AP may be related to the activity of NMDA receptors. Thus, we decided to focus on the low-dose dizocilpine model to investigate the production of NOS1AP under NMDAR inhibition in vivo.

**Figure 1:**
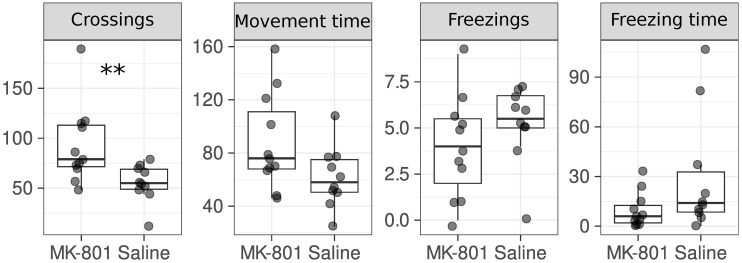
Injections of MK-801 led to an increase in the number of crossings. The following parameters of rats were measured in the open field test: number of crossings, movement time (s), number of freezings, freezing time (s). Among the behavioral tests, there were also the number of stands (with and without support), the number of boluses, the number of urinations, and the number of snorkels and tail lifts. The number of points corresponds to the number of animals. **—*p*-value < 0.01 (Wilcoxon Rank-Sum Test).

We reproduced the increased locomotor activity of rats after dizocilpine injections (Figure 1). In addition to the previous work [19], we also analyzed other parameters of rats’ activity in the open field test, but no differences were found. We also monitored the animals’ body weight throughout the study and observed no effects of MK-801 on this parameter.

**Figure 2:**
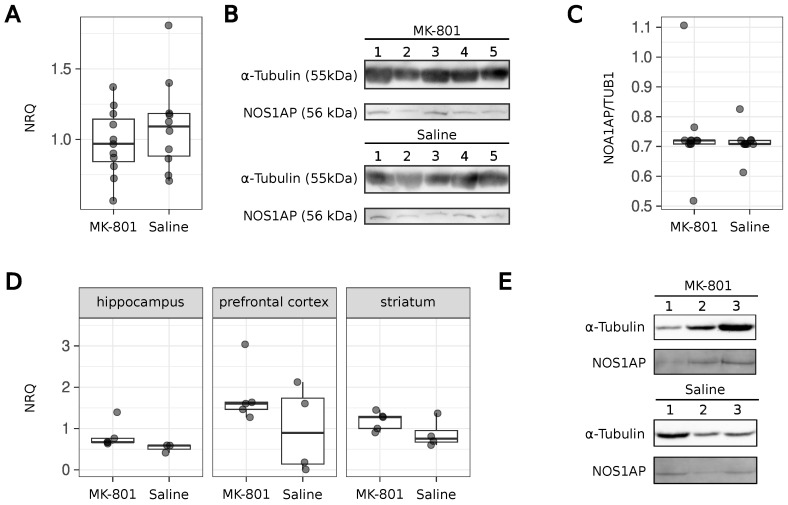
*NOS1AP* gene expression and the production of NOS1AP protein are not altered in rat brains with schizophrenia symptoms induced by dizocilpine injection. (**A**)—qRT-PCR results for cDNA isolated from rat brain. Animals were subjected to series of MK-801 injections during 6 days (0.1 mg/kg). *ACTB* and *RPLP0* genes were used to calculate the normalized relative quantity (NRQ). (**B**)—Western blotting results of protein lysates isolated from animal brains. Anti-NOS1AP and Anti-α-Tubulin antibodies were used. Bands for five samples from each group are presented. The samples presented were analyzed on the same gel. (**C**)—The ratio of NOS1AP protein signal to α-Tubulin signal in the group of rats injected with dizocilpine and in the control group. (**D**)—qRT-PCR results for cDNA isolated from different parts of the rat brain. The same genes were used to calculate NRQ. (**E**)—Western blotting results of protein lysates isolated from the prefrontal cortex. The same antibodies were used (see the description of panel **B**).

We conducted two sets of experiments on rats. In the first case, the sizes of the experimental and control groups were six and five animals, respectively; in the second case, there were five animals in each group. After verifying the presence of behavioral changes, we compared the *NOS1AP* gene expression and NOS1AP protein production in these animals. RNA and proteins were isolated from the medial prefrontal cortex, hippocampus, and striatum. The literature reports that NOS1AP is produced in these parts of the brain [21,22]. Our experiments showed that *NOS1AP* gene expression and NOS1AP protein production were not altered in response to MK-801 injections (Figure 2A–C). These data were collected from the samples containing several brain parts. To verify that we had not missed any specific effects, we repeated the animal experiments for groups of five animals and collected the prefrontal cortex, striatum, and hippocampus separately. However, no changes in the RNA level of *NOS1AP* were observed (Figure 2D). The level of the corresponding protein was unchanged in the samples from the prefrontal cortex (Figure 2D). For other lysates, we could not detect the signal from Anti-NOS1AP antibodies, likely due to their low sensitivity.

In vitro models are an important tool for studying disease mechanisms and potential pharmacological treatments. Advances in cell culture techniques can help in understanding neuropsychiatric disorders. The human neuroblastoma cell line SH-SY5Y is most commonly used to study schizophrenia. Cells from this line exhibit biochemical and functional characteristics of neurons, such as neurite outgrowth and neurotransmitter synthesis. This cell line synthesizes specific proteins and protein isoforms that are not present in primary rodent cultures [37]. MK-801-treated SH-SY5Y cells can be used as a model of neuronal dysfunction to study schizophrenia [38,39]. The ancestor of HEK293T (HEK293) has been shown to exhibit many of the characteristics of undifferentiated neurons [40]. This cell line has also been used in studies of schizophrenia [41]. Thus, HEK293T may also be a suitable neuronal model. These cells expressed *NOS1AP* (see results below).

**Figure 3:**
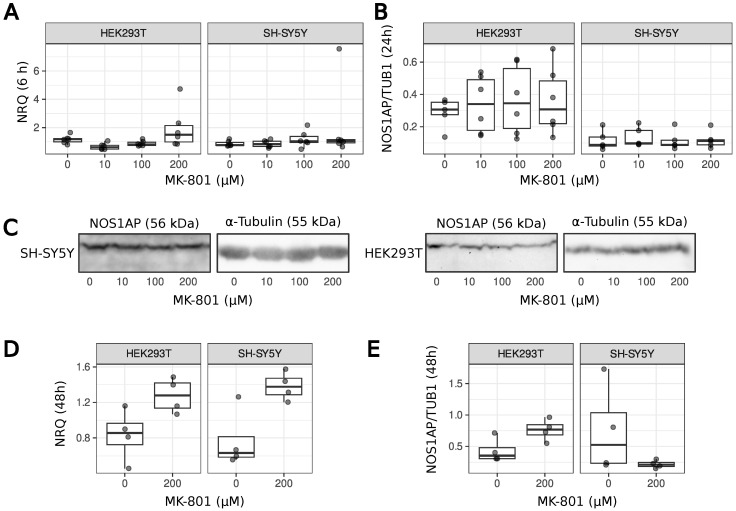
*NOS1AP* gene expression and the production of NOS1AP protein are not altered in cells treated with MK-801. (**A**)—qRT-PCR results for cDNA isolated from SH-SY5Y and HEK293T cells. *ACTB* and *RPLP0* genes were used to calculate the normalized relative quantity (NRQ). Cells were collected after 6 h of treatment. (**B**)—The ratio of NOS1AP protein signal to α-Tubulin signal in the Western blotting results of protein lysates from mammalian cells. Typical results are presented in the panel. (**C**)—Anti-NOS1AP and Anti-α-Tubulin antibodies were used. Cells were collected after 24 h of treatment. The samples presented were analyzed on the same gel. (**D**)—qRT-PCR results for cDNA isolated from SH-SY5Y and HEK293T cells after 48 h of treatment. (**E**)—The ratio of NOS1AP protein signal to α-Tubulin signal in the Western blotting results of protein lysates from mammalian cells after 48 h of treatment.

We also analyzed the change in *NOS1AP* gene expression and NOS1AP protein production in SH-SY5Y neuroblastoma cells and HEK293T after MK-801 treatment. In the experiment on animals, we used only one low-dose treatment, and the results obtained can only describe a specific model. To be able to at least speculate about the effect of MK-801 on NOS1AP production in other schizophrenia models (high dose of MK-801, from 10 to 200 µM), we used different concentrations of the dizocilpine in cell line experiments, where 10 µM provides a blockade of NMDAR and 200 µM is the maximum concentration used in the literature [41]. In agreement with the previous results, no differences in *NOS1AP* expression and protein levels were found in both cell lines (Figure 3), even after 48 h of treatment (Figure 3D,E). Following long-term treatment, it can only be supposed that there is a slight trend towards an increase in *NOS1AP* mRNA but not protein levels. Based on the combined data, we can conclude that MK-801 does not affect NOS1AP production in vitro or in vivo.

## 4. Discussion

We have previously shown that NOS1AP is capable of forming stable aggregates in yeast and mammalian cells [30]. Increased levels of *NOS1AP* mRNA, as well as the corresponding protein, have been reported in patients with schizophrenia [13]. This led us to speculate that NOS1AP overproduction in schizophrenia may lead to the formation of NOS1AP aggregates [30]. We therefore hypothesized that NOS1AP aggregates are able to sequester nNOS. This may lead to a decrease in the number of nNOS complexes with NMDA receptors, reducing NMDA-mediated calcium influx and deactivating the catalytic activity of nNOS. The reduced activity of these receptors is thought to be associated with the development of schizophrenia (for review, see [15]). From another point of view, the activation of NMDAR affects the production of NOS1AP [17], but there are no data on the effect of inactivation of this signaling pathway. Based on this, we wanted to test whether an overproduction and aggregation of the NOS1AP protein occurs in schizophrenia and, in particular, under NMDAR inhibition in vivo and in vitro.

Several non-competitive NMDA receptor antagonists have been described and used, such as dizocilpine (MK-801). Compared to other NMDA receptor antagonists such as phencyclidine and ketamine, MK-801 has a more potent inhibitory effect on NMDA receptors, with a higher affinity and specificity [42,43]. It is noteworthy that a proteomic study showed alterations in the production of several proteins (HSP60, HSP72, NSE, DRP-2, etc.) in the thalamus of rats subjected to repeated MK-801 injections [44].

A number of animal models based on MK-801 have been described in the literature, varying in dose (from 0.1 to 0.5 mg/kg) and their duration of treatment [19,36,41,45,46]. In our work, we have focused on the low-dose models [19] that lead to an increase in locomotor activity in animals and affect glutamate and glutamine levels. This model rather reflects the hypofunction of the NMDAR, but allows the production of NOS1AP to be studied under such conditions. Animals subjected to the MK-801 injections demonstrated increased locomotor activity as previously reported [19]. This led us to believe that the animals should also share other molecular phenotypes, such as changes in glutamate and glutamine levels. For the first time, we have shown that low doses of MK-801 do not lead to other behavioral changes seen in the open field test (Figure 1). MK-801 does not induce changes in NOS1AP production in animals (Figure 2) or cell cultures (Figure 3). We can only suppose a slight trend towards an increase in NOS1AP mRNA, but not protein, levels during long-term treatment with high MK-801 concentrations. Therefore, we cannot completely exclude the possibility that more acute treatment may lead to changes in NOS1AP expression. An increase in NOS1AP production has been described in patients with schizophrenia [13]. From this point of view, our results may illustrate the potential limitations of using pharmacological models with low doses of MK-801 to mimic some of the molecular changes that occur in schizophrenia. As we have only tested one animal model [19], we cannot conclude that other dizocilpine treatments (e.g., at higher doses) will not affect NOS1AP levels. However, we have shown that MK-801 has no effect on *NOS1AP* expression in a wide range of concentrations in two cell lines (Figure 3). In conclusion, dizocilpine treatment does not affect NOS1AP expression in vitro and at low doses (0.1 mg/kg) in rats in vivo.

## Figures and Tables

**Table 1:** Primers used in this work. Primer sequences for the β-actin gene and *RPLP0* originated from [24]. Primers efficiencies were calculated according to previously published recommendations [25].

Primer	Sequence	Annealing T (°C)	Primer Efficiency (%)
NOS1AP_1&3i_F	GAAAGTGATTCTGAAGAAGAAG	55.7 and 62.0	101
NOS1AP_R	CGATTCTCATAGCTTGGC	55.7 and 62.0	101
b-actin_F	CATCCTCACCCTGAAGTACCC	55.7	100
b-actin_R	CTCAAACATGATCTGGGTCATCTT	55.7	100
RPLP0_F	CAACCCAGCTCTGGAGA	62.0	114
RPLP0_R	CAGCTGGCACCTTATTGG	62.0	114

## Data Availability

The raw data are presented in figues and are available upon request.

## Abbreviations

The following abbreviations are used in this manuscript:

FBS	Fetal bovine serum
MK-801	Dizocilpine
NMDA	N-methyl-D-aspartate
NMDAR	NMDA receptor
NRQ	Normalized relative quantity
NOS1	Nitric Oxide Synthase 1
NOS1AP	Nitric Oxide Synthase 1 Adapter Protein
PDZ-BD	PDZ-binding domain
PTB	Phosphotyrosine-binding domain
RPLP0	Ribosomal Protein Lateral Stalk Subunit P0
SNVs	Single-nucleotide variations
